# Research on the Surface-State Parameterization of a Refill Friction Stir Spot Welding Joint Made of Aluminum Alloy and Its Connection to the Fracture Mode

**DOI:** 10.3390/ma17030762

**Published:** 2024-02-05

**Authors:** Hua Zhong, Guocheng Xu, Juan Dong, Xiaopeng Gu, Qiuyue Fan

**Affiliations:** 1 Department of Materials Science and Engineering, Jilin University, Changchun 130025, China; zhonghua202402@163.com (H.Z.);; 2 Fujian Provincial Key Laboratory of Welding Quality Intelligent Evaluation, Longyan University, Longyan 364012, China

**Keywords:** surface testing, refilled friction stir spot welding, quality evaluation, support vector machine

## Abstract

Surface features are crucial for assessing welding quality because they serve as an intuitive depiction of the quality of the joint and have a major influence on welding strength. According to the characteristics of the refill friction stir spot welding (RFSSW) process and an analysis of the surface-state and internal morphology of RFSSW joints, a method of predicting the mechanical properties of RFSSW joints based on surface-state characteristics was proposed. In this paper, a laser-ranging sensor was used to characterize the surface state of RFSSW joints, and parametric characterization methods of the surface-state features of RFSSW joints were proposed. On this basis, a support vector machine was used to predict and analyze the fracture mode of RFSSW joints. The accuracy of the analysis of the test samples reached 95.8%. This paper provides a more efficient and convenient new method for the quality evaluation of RFSSW joints.

## 1. Introduction

The refill friction stir spot welding (RFSSW) method is essentially a solid-phase welding technique in which the welding temperature is lower than the melting point of the material [1,2]. Due to its significant advantages of a small heat-affected zone and microstructure deformation, as well as small welding deformation, it has better application prospects in the fields of aerospace, automotive manufacturing, shipbuilding, and other industries [3,4,5]. The quality of RFSSW joints directly affects the reliability of the structure and the service safety of the joint. At present, the quality evaluation of RFSSW joints mainly adopts two technical paths based on welding process parameters or non-destructive testing. The evaluation of joint quality based on welding process parameters mainly employs the response surface method and machine learning technology to predict the joint quality through various welding process parameters [6,7,8]. RFSSW involves welding process parameters such as the insertion depth of the sleeve, stirring speed, stirring time, and refilling time. Relevant studies have been carried out. For example, de Castro et al. [9] explored the effects of tool rotational speed, welding time, and tool plunge depth on a joint’s strength using the Taguchi approach. Santana et al. [10] applied the response surface method to establish a regression equation of stirring speed and plunge depth to predict the mechanical properties of joints. Birsan et al. [11] utilized Neural Networks based on plunge depth, stirring speed, and residence time to predict the maximum temperature and stress values of joints. However, considering only the influence of welding process parameters, the influence of the objective environment on welding quality is often overlooked, such as tool wear, mechanical assembly errors, and other factors. This leads to significant prediction errors in the quality assessment of joints based on welding process parameters, and the use of non-destructive testing to characterize the structure and defects of joints can more accurately evaluate the quality of joints.

Non-destructive testing methods are divided into internal and surface testing. Internal testing methods include X-ray testing and ultrasonic testing [12,13], while surface testing includes magnetic particle testing and penetration testing, and so on. Different testing methods are suitable for different applications. The characteristics and defects of RFSSW joints include effective connection area, voids, incomplete refill defects, etc. Currently, research on RFSSW joint quality based on non-destructive testing results exists in surface testing and ultrasonic testing. Schmal et al. [14] utilized C-scan images, which were produced by ultrasonic testing, to evaluate the connection status of RFSSW joints. They concluded that the bearing capacity under quasi-static lap shear and cross-tensile loads was correlated with the testing results. Li et al. [15] used ultrasonic testing images combined with three-dimensional feature to evaluate shear strength, but the ultrasonic detection method had high requirements for the test conditions. Ahmed et al. [16] performed image segmentation and feature extraction on joint images to identify defects such as surface cracks, gas inclusions, etc., and to analyze the relationship between friction and surface defects. However, their research lacks an evaluation of the mechanical properties of joints. The most popular research direction in surface testing is mainly image detection [17,18]. This method can quickly quantitatively analyze the surface defects of a joint. However, the defects on the surface alone are not sufficient to evaluate the quality of the joint.

Due to the unique welding process of RFSSW, the geometric shape of the joint not only reflects the joint characteristics but also reflects the defect characteristics. Therefore, the quantitative evaluation of the surface structure of RFSSW joints can better reflect the joint quality. Therefore, this paper proposes a quality evaluation method for RFSSW based on surface testing. This method employs surface testing technology based on a laser-ranging sensor, which offers the advantages of high testing accuracy, digital testing results, safety, and reliability [19,20,21]. It has been used to test and analyze the surface of aluminum alloy RFSSW joints. The relationships between surface characteristics such as surface roughness, dents and flashes, and joint mechanical properties were studied to predict the quality of an RFSSW joint. This rapid and convenient quality testing method for RFSSW joints based on a laser-ranging sensor can quickly identify unqualified joints in industrial production to ensure quality and safety.

## 2. Materials and Methods

### 2.1. Welded Material and Preparation of the Specimens

**Figure 1:**
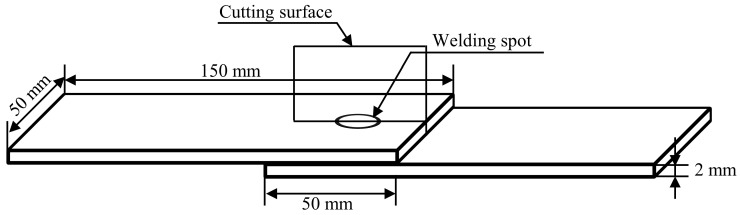
Diagram of an RFSSW specimen.

**Figure 2:**
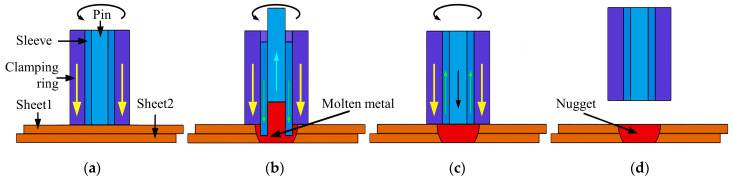
RFSSW processes: (**a**) push-down and tool rotation; (**b**) sleeve stir and pin pullback; (**c**) sleeve pullback and pin stir; (**d**) pressure relief.

**Figure 3:**
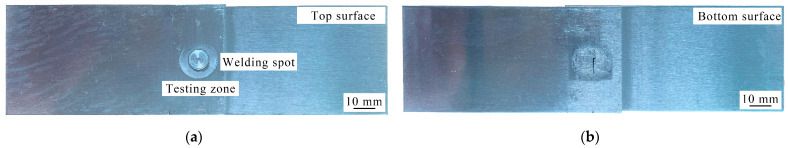
Photographs of an RFSSW specimen: (**a**) the top surface of the specimen; (**b**) the bottom surface of the specimen.

The chemical composition of the welded material commonly used for the manufacture of vehicles and railways with the aluminum alloy material EN AW-5083 is shown in Table 1. The dimensions of the tested plates were 150 mm × 50 mm × 2 mm. The specifications of the tension specimens of the RFSSW joints are shown in Figure 1. The welding spot was located in the middle of the overlap zone of the specimen and the metallographic specimen of the RFSSW joint was prepared by taking a cross-section of the joint through the cutting surface. The RFSSW specimens were prepared by an HT-FSW500MT stir friction spot welding machine (Shanghai Aerospace Equipment Manufacturing Plant Aerospace Engineering Equipment (Suzhou) Co., Ltd., Shanghai, China), and the tool system consisted of an 18 mm clamping ring, 9 mm sleeve, and 5 mm pin. The RFSSW process is demonstrated in Figure 2 [11]. The experimental welding parameters selected in this paper are shown in Table 2. Figure 3 shows photographs of an RFSSW specimen. Figure 3a shows the top surface of the RFSSW joint; this surface was selected as the laser scanning testing surface.

### 2.2. Surface Testing of the RFSSW Joint Based on the Laser Ranging and Imaging Method

**Figure 4:**
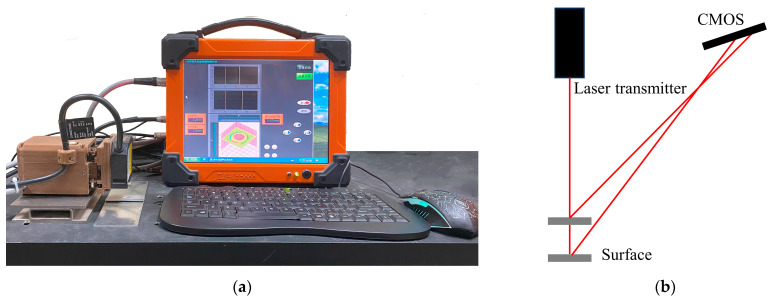
The RFSSW joint surface scanning testing device: (**a**) testing device; (**b**) the detection principle of the laser-ranging sensor.

**Figure 5:**
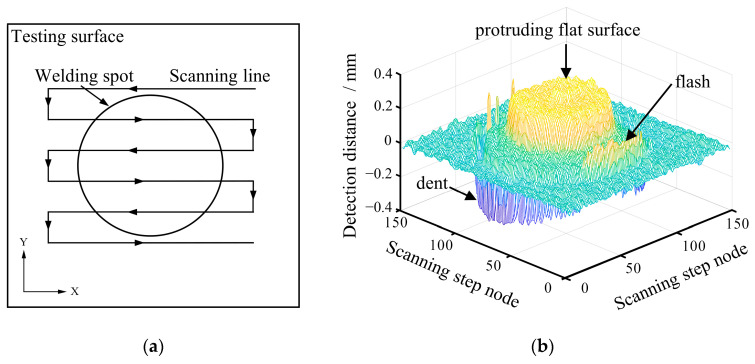
Schematic diagram of the detection path and result of the RFSSW joint: (**a**) surface scanning testing method; (**b**) stereoscopic testing image.

The RFSSW joint surface scanning testing device based on a laser-ranging sensor is shown in Figure 4a. The device consists of a computer, a laser-ranging sensor, and a scanning mechanism. The laser-ranging sensor is a Panasonic HL-G103-A-C5 and it projects the laser beam to the surface of the measured object and receives the laser reflected by the surface of the measured object. According to the principle of triangulation, the movement of the reflected laser in the sensor is converted into a displacement signal, and the distance between the sensor and the measured object is calculated, as shown in Figure 4b. The scanning mechanism drives the laser-ranging sensor to scan the scanning path on the surface of the RFSSW joint, as shown in Figure 5a. This scanning method was used to scan the RFSSW joint surface in the X/Y testing plane with a step length of 80 μm in the X and Y directions, as shown in Figure 5a. According to the size of the RFSSW joint, the scanning zone was set at 12 mm × 12 mm. The laser-ranging sensor obtains the testing distance value at each mechanical scanning stepper node and corrects the RFSSW joint’s testing result by setting the joint edge’s testing distance value to the datum plane, as shown in Figure 5b. The three-dimensional image formed by the distance data obtained from all the scanned testing points can express the features of the RFSSW joint, such as flashes and dents, and can be quantitatively analyzed based on pixel data. To reduce the interference of the electrical noise from the testing instrument and other factors, the obtained digital image was denoised by a Butterworth lowpass filter so that the surface topography characteristics of the RFSSW joint were clearer and the analyzed data were more accurate.

## 3. Results

### 3.1. Analysis of the Relationship between the Surface State and Quality of the RFSSW Joint

**Figure 6:**
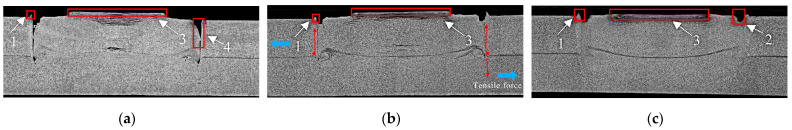
Metallographs of typical RFSSW joints and fracture mode diagram: (**a**) Group 7; (**b**) Group 12; (**c**) Group 15.

In this paper, three typical RFSSW joints were selected to analyze the relationship between the surface state and quality of the RFSSW joints. The cross-section metallographs of the RFSSW joints are shown in Figure 6. In the RFSSW process, the molten metal enters the gaps between the clamping ring, sleeve, and pin during the welding process, so the surfaces of all the specimens exhibit obvious welding marks, as shown in Figure 3a.

Since the sleeve and the pin move vertically in opposite directions during the welding process, if the pin and the sleeve can be aligned and made parallel to the surface of the welded specimen after refilling, the center of the RFSSW joint will be relatively flat, as shown in zone 3 in Figure 6c. If the pin and the sleeve are not perfectly aligned and parallel to the surface of the welded specimen, a protruding flat surface corresponding to the diameter of the pin will appear, as shown in zone 3 in Figure 6a,b. Since the clamping ring is fixed when the sleeve rotates and moves downward, most of the molten metal enters the cavity between the pin and the sleeve, and a small part enters the gap between the sleeve and the clamping ring to form a flash, as shown in zone 1 in Figure 6a–c. When the sleeve is rotated upward for refilling, the extruded metal will not be completely refilled, and there will be a dent on the edge of the stirring zone of the sleeve, as shown in zone 2 in Figure 6c. When the joint has incomplete refilling defects, it will also appear as a dent on the edge of the stirring zone of the sleeve, as shown in zone 4 in Figure 6a. Therefore, when improper welding parameters lead to voids and incomplete refilling defects, there may be different surface states such as too much extruded metal. These surface-state characteristics reflect the internal quality of the RFSSW joint and its mechanical properties, laying the foundation for quality evaluation based on the surface-state characteristics of RFSSW joints.

### 3.2. Calculation of the Surface Characteristic Parameters of the RFSSW Joints

The surface characteristics of RFSSW joints mainly include bumps, dents, and roughness. In this paper, based on the digital image information obtained from the surface testing of RFSSW joints, the surface characteristics of the RFSSW joints are parameterized to study the relationship between the surface state of an RFSSW joint and its mechanical properties. To achieve parametric quantitative analysis of the surface characteristics of the RFSSW joints, this paper uses the volume of metal extruded from the surface to characterize the convex state of the surface of the RFSSW joint, uses the concave depth of the surface of the joints, the sum of the height of the flash, and the depth of the dent to characterize the convexness on the surface, and calculates and characterizes the surface roughness according to the general method. The surface of the base material zone of the RFSSW joint is considered the datum plane, and the metal extrusion volume is the difference between the volume of the bumps above the datum plane and the volume of the dents below the datum plane, that is, the net extruded metal volume.

**Figure 7:**
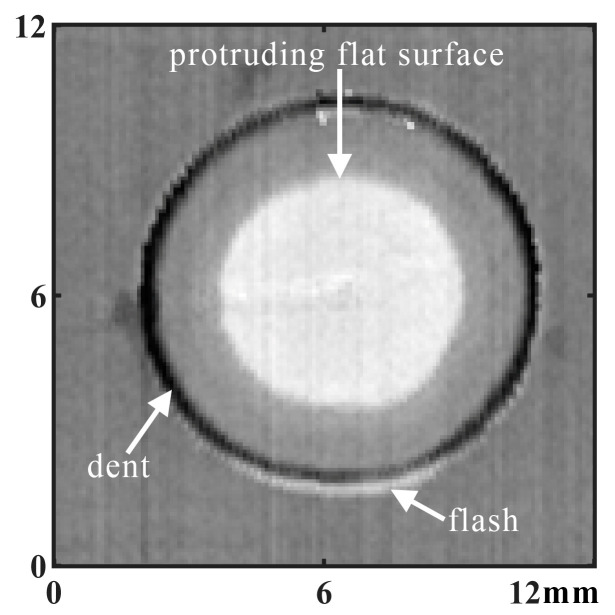
Two-dimensional surface testing image of an RFSSW joint.

To facilitate the analysis, the topography of the surface of the RFSSW joint was converted from Figure 5b to the image shown in Figure 7. In Figure 7, the black zone corresponds to the dent in the stirring zone of the sleeve, and the light-colored zones correspond to the protruding flat surface in the stirring zone of the pin and the flash around the joint. 

The formula for calculating the net extruded metal volume *V* on the surface of the RFSSW joints is shown in Equation (1):

(1) $$V=\Delta S\times \sum_{i=1}^{n}y_{i}$$

where *y_i_* is the grey value of the pixel in the image, that is, the convex or concave state of the corresponding position on the surface of the RFSSW joint; *y_i_* is positive when it is convex and negative when it is concave; and ΔS is the area represented by each pixel, which is related to the scanning step of surface testing. According to the scanning step size used in this paper, the area is 6.4 × 10^3^ µm^2^. Based on the volume conservation law in material plastic deformation [22], the calculation result of Equation (1) can reflect the overall net extruded metal volume of the RFSSW joints, according to which the RFSSW joint can be evaluated to determine whether voids and incomplete refilling defects exist. When the amount of metal extruded from the RFSSW joint is large, the degree of the internal voids and incomplete refilling defects is also large.

**Figure 8:**
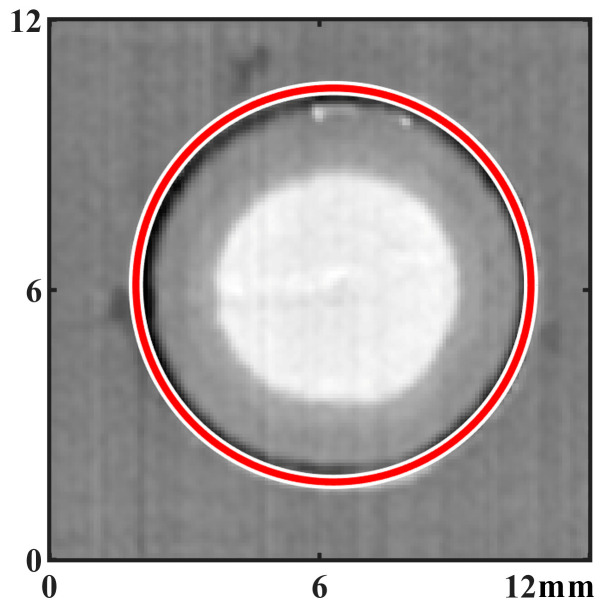
Surface testing to identify the RFSSW joint dent zone.

**Figure 9:**
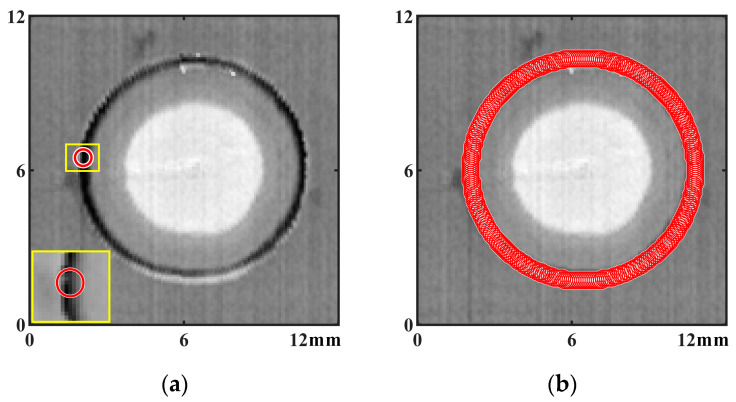
The method of selecting the circle zone for calculating the concavity of the surface: (**a**) schematic diagram of a circle zone used for calculation; (**b**) distribution of all the circle zones used for calculation.

Since dents and flashes on the surface of RFSSW joints are associated, the methods used in this paper to calculate the parameters of dents are as follows: First, pixels that represent dents are identified through Hough circle transformation analysis on the digital image of the RFSSW joint, as shown in Figure 8 [23], and then, a circular zone with a radius of 0.32 mm is selected with each concave pixel as the center, as shown in Figure 9a. As shown in the partially enlarged image, the circular zone contains both the flash and the dent of the RFSSW joint. For the circular zone set by each pixel on the Hough circle in Figure 9b, the maximum flashheight *D_flash_* and maximum dent depth *D_dent_* in each circular zone are analyzed and calculated, and the calculation formulas are shown in Equation (2) and Equation (3), respectively. The sum of the bump height and dent depth *D_flash-dent_* is calculated by Equation (4). Finally, *D_flash_*, *D_dent_*, and *D_flash-dent_* are obtained through all the pixels in the circular zone. The average and maximum values of *D_dent_* and *D_flash-dent_* obtained from all the circular zones are calculated, representing the characterization parameters of the ultimate states and distribution characteristics of the flashes and dents on the surface of the RFSSW joint. These parameters strongly correlate with incomplete refilling defects and voids in the RFSSW joints.

(2) $$D_{flash}=\max\left(y_{1},y_{2},\ldots ,y_{n}\right)$$

(3) $$D_{dent}=\left|\min\left(y_{1},y_{2},\ldots ,y_{n}\right)\right|$$

(4) $$D_{flash-dent}=D_{flash}+D_{dent}$$

**Figure 10:**
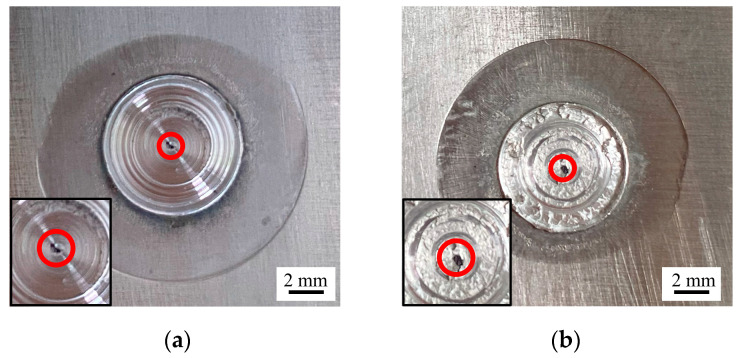
Actual RFSSW joint photographs: (**a**) smooth surface; (**b**) rough surface.

A parametric calculation method of the surface roughness of RFSSW joints is described here. As shown in the photographs of the RFSSW joints in Figure 10, most of RFSSW joints have a smooth surface in the pin stirring zone, while a few RFSSW joints are rough. Since the surface features of the RFSSW joints, such as flashes and dents, are characterized by Equations (1)–(4), the calculation of the surface roughness of the RFSSW joints in this paper mainly focuses on the central pin stirring zones of the joints, represented by the red circles shown in Figure 10, and the calculated roughness reflects only the effect of the pin stirring on the surface metal of the joints. The calculation formula for the surface roughness *Ra* is shown in Equation (5):

(5) $$Ra=\frac{\sum_{i=1}^{n}\left|y_{i}-y_{m}\right|}{n}$$

where *y_i_* is the grey value of each pixel of the testing image corresponding to the calculation zone, that is, the height of the convex part and the depth of the concave part; *y_m_* represents the average value of all pixels detected in the pin stirring zone; and *n* is the number of pixels involved in the calculation zone. The larger the value of *Ra*, the rougher the surface of the RFSSW joint.

### 3.3. Study of the Correlation between the Surface Characteristic Parameters and Mechanical Properties of RFSSW Joints

**Figure 11:**
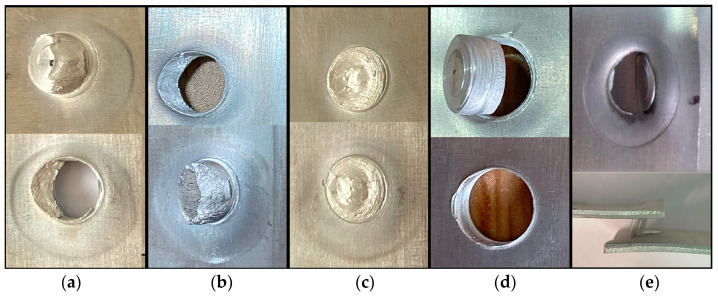
The fracture modes of the RFSSW joints are as follows: (**a**) plug shear fracture along the upper plate; (**b**) plug shear fracture along the lower plate; (**c**) shear fracture; (**d**) plug-type fracture; (**e**) base metal fracture.

**Figure 12:**
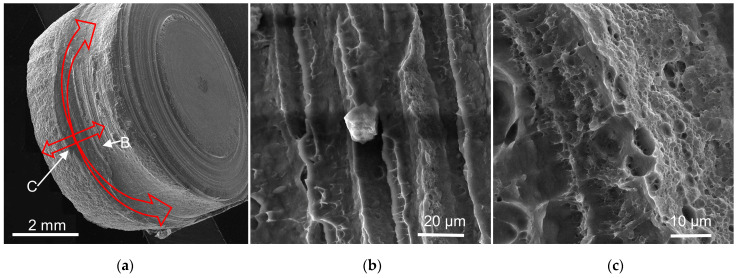
Scanning electron microscopy images of plug-type fracture: (**a**) whole nugget; (**b**,**c**) magnified views of regions B and C marked in (**a**).

The mechanical properties of RFSSW joints are generally determined by the tensile fracture mode and tensile strength of the joints. Through experiments, it was observed that the RFSSW joints exhibited four fracture modes: plug shear, shear, plug-type, and base metal fracture, among which plug shear fracture was divided into two modes, a fracture along the upper plate and a fracture along the lower plate, as shown in Figure 11. Among all the fracture modes, the plug shear, shear, and base metal fracture modes generally occur at the exit line of the sleeve, in the coated aluminum layer, and in the base material zone, indicating that the mechanical properties of the RFSSW joints are good [24,25,26]. A plug-type fracture is caused by more incomplete refilling defects or voids in the border zone of the joints because of poor metal fluidity during the process of stirring. The fracture mechanism was discussed by Sun et al. and Zou et al. [26,27]. The plug-type fracture breaks completely at the boundary of the joint, resulting in low tensile strength and poor mechanical properties. During the tensile testing of the RFSSW specimens, the stress concentration generated at the edge of the nugget easily causes cracks to spread along fewer effective connection zones due to incomplete refilling defects and holes, as shown in Figure 6b. The cracks spread along the annular boundary until the joint breaks. Figure 12a shows that the button morphology completely pulled away from the RFSSW joint. At the upper plate of the joint, the fracture morphology of the joint exhibits a striation pattern, as shown in Figure 12b, due to the voids, incomplete refilling defects, and weak connection surface. At the lower plate of the joint, a ductile fracture morphology is observed, as shown in Figure 12c, indicating that the lower plate is well connected.

**Figure 13:**
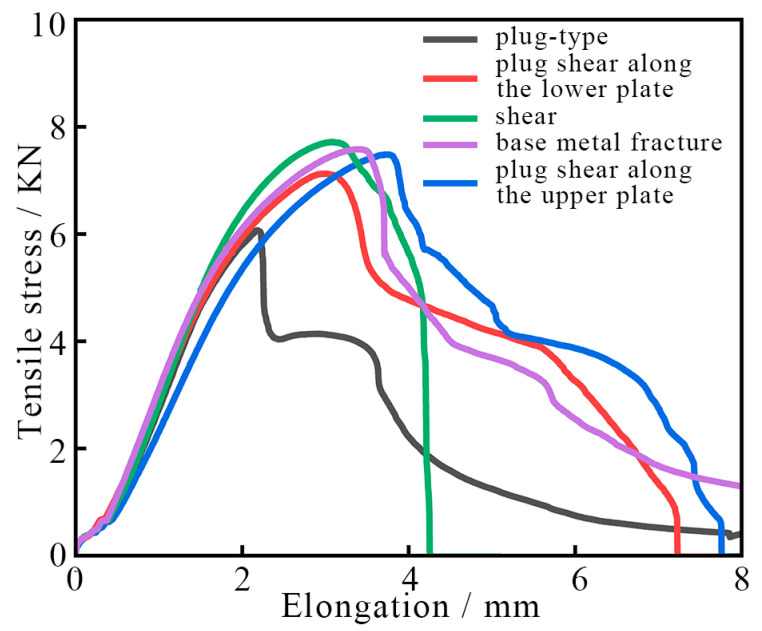
Comparison diagram of the tensile curves corresponding to different RFSSW joint fracture modes.

The tensile curves corresponding to various fracture modes of the RFSSW specimens are shown in Figure 13. As shown in Figure 13, for the plug-type fracture mode, when the tension force reaches approximately 6 kN, the crack first occurs at the weak connection boundary of the RFSSW joint; then, the crack expands rapidly, and the tension force drops to approximately 4 kN. Then, the crack spreads to the lower plate and finally produces a ductile fracture. Compared with the plug-type fracture mode, a ductile fracture or brittle fracture occurs after the tension forces of the RFSSW joint specimens with other fracture modes reach 7 kN. In comparison, the plug-type fracture can bear less tensile strength, and the fracture speed is faster, so the harm is greater; moreover, such a failure mode needs to be detected and discovered in time.

**Figure 14:**
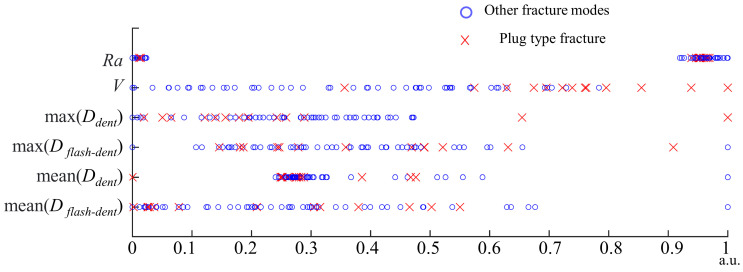
Relationship between the fracture modes and surface characteristic parameters of the RFSSW joints.

Based on the test data of 64 specimens, the correlation between the fracture mode under tensile forces and the surface characteristic parameters of RFSSW joints is studied in this paper. Figure 14 shows the test results of the relationships between the fracture mode and the surface characteristic parameters of the RFSSW joints. In the figure, the horizontal axis coordinate represents the normalized surface characteristic parameter values, and special symbols are used to represent the fracture modes of the RFSSW joint specimens with different surface characteristic parameter values. Figure 14 indicates that some surface feature parameters have a strong linear correlation with the fracture mode, while some feature parameters have a weak or even no linear correlation with the fracture mode, and the correlations between each feature parameter and the fracture mode are quite different. Among the surface characteristic parameters of the RFSSW joints, the net extruded metal volume *V* has the strongest correlation with the fracture mode. With increasing *V*, the probability of a plug-type fracture increases obviously. Other surface feature parameters, such as the maximum *D_dent_*, the roughness *Ra*, and the maximum *D_flash-dent_*, have a weak correlation with the fracture mode, but accurate prediction of the joint fracture mode cannot be achieved by any single feature parameter. 

## 4. Discussion

**Figure 15:**
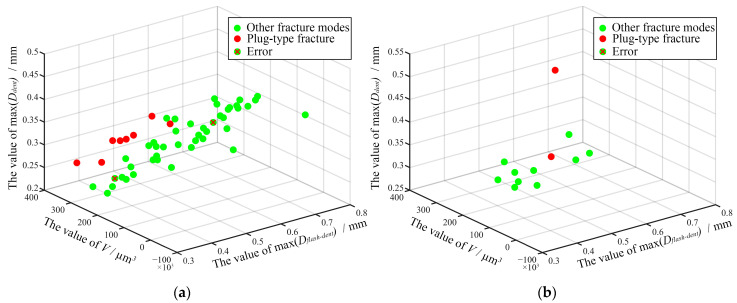
The results of SVM classification: (**a**) training set; (**b**) testing set.

This paper adopted a support vector machine (SVM) model that supports the prediction of the binary classification of small-sample data and predicts the fracture modes of RFSSW joints based on multiple surface characteristic parameters [18,28]. The test data of 64 RFSSW joints were randomly divided into two groups: one group accounting for 80% of the total data set was used to train the SVM, and the other group accounting for 20% of the total data set was used to test the performance of the SVM. Kernel functions such as linear, quadratic, and polynomial functions were used for classification analysis of the SVM, and the performance of each kernel function was measured using the k-fold cross-validation technique with a fold number of 5. The classification accuracy of the different kernel functions is shown in Table 3. The classification accuracy of the linear kernel function is higher, up to 85.9%, while the accuracy of the SVM optimized by the Bayesian optimization is even higher, up to 95.8%. To improve the efficiency and reduce the calculation time, surface feature parameters that are extremely weakly related to the fracture mode were deleted for dimensionality reduction. Only three surface feature parameters, the net extruded metal volume *V*, the maximum *D_flash-dent_*, and the maximum *D_dent_*, were used for fracture mode classification analysis via SVM with Bayesian optimization. The accuracy can reach 95.8%, and the classification results of the training set are shown in Figure 15a. The SVM was tested by using the data of 12 specimens in the test set, and the plug fracture modes were correctly detected, as shown in Figure 15b.

## 5. Conclusions

1. The experimental analysis revealed that due to the particularity of the RFSSW process, the surface characteristics of RFSSW joints reflect the internal defects and connection states of the joints to a certain extent, and thus, are correlated with the mechanical properties of the joints.
2. The surface-state image characterization method of RFSSW joints based on the surface detection of laser ranging was studied, and methods for the parametric characterization of the surface-state features of RFSSW joints are proposed, laying a foundation for the prediction of the mechanical performance of an RFSSW joint based on the surface characteristics of the joint.
3. Based on the surface characteristic parameters of RFSSW joints, an SVM was adopted to classify and analyze the fracture modes of the RFSSW joints, with an accuracy of 95.8%.

The surface testing method proposed in this paper can rapidly identify potentially hazardous plug-type fractures in RFSSW joints. Notably, this approach outperforms other non-destructive testing techniques in terms of efficiency. It is essential to note that this method is mainly suitable for use on RFSSW joints that are directly exposed to the laser beam. Therefore, it has limited application in zones that are shielded or for other types of joints. This paper focuses on post-welding non-destructive testing and quality assessment methods for RFSSW joints, without delving into the research of non-destructive testing technology during the welding process. The relationship between the heat input in the welding process and the quality of the joints is the research direction that should be taken for real-time non-destructive testing.

## Figures and Tables

**Table 1:** Chemical composition of EN AW-5083 (wt.%).

Cu	Si	Fe	Mn	Mg	Zn	Cr	Ti	Al
0.1	0.4	0.4	0.4–1.0	4.0–4.9	0.25	0.05–0.25	0.15	Bal.

**Table 2:** Experimental welding parameters for EN AW-5083.

Group	Stirring Time (s)	Depth (mm)	Rotational Speed (rpm)	Refilling Time (s)
1	2.5	2.4	1000	1.5
2	2.5	2.4	1000	2
3	2.5	2.4	1000	2.5
4	2.5	2.4	1000	3
5	2.5	2.4	1400	1.5
6	2.5	2.4	1400	2
7	2.5	2.4	1400	2.5
8	2.5	2.4	1400	3
9	2.5	2.4	1800	1.5
10	2.5	2.4	1800	2
11	2.5	2.4	1800	2.5
12	2.5	2.4	1800	3
13	2.5	2.4	2200	1.5
14	2.5	2.4	2200	2
15	2.5	2.4	2200	2.5
16	2.5	2.4	2200	3

**Table 3:** Classification accuracy of different kernel functions.

Kernel Function	Accuracy
Linear	85.9%
Quadratic	73.4%
Polynomial	78.1%
SVM with Bayesian optimization	95.8%

## Data Availability

Data are contained within the article.
